# U.S. Military Medical Surveillance: Two Centuries of Progress

**Published:** 2025-04-20

**Authors:** Sanders Marble

**Affiliations:** U.S. Army Medical Department Center of History and Heritage, U.S. Army Medical Center of Excellence, San Antonio, TX

The U.S. Army began coordinated medical surveillance over 200 years ago. The earliest records of the U.S. military medical corps are incomplete, in no small part due to the British burning of official buildings in Washington in 1814, but it is verifiable that regular reporting of medical information about Army personnel occurred at least as early as 1814. ^1^

In 1818, following post-War of 1812 reorganization of the U.S. Army, its first official Surgeon General, Joseph Lovell, ordered all Army surgeons to regularly report on the diseases they treated. Those early medical reports ordered by Lovell were completed monthly, then compiled and sent quarterly to Washington, DC. With horses the fastest, but exhaustible, means of communication, there was no chance of prompt response to news of an outbreak in any remote area. Disease monitoring had severe limits, because treatment was limited by the communication technologies of the time. Even if information could be advanced relatively rapidly, sick patients transported over dirt roads to hospitals with no better diagnostic tools nor treatment methods would likely experience worse outcomes. Coastal forts and posts in the eastern seaboard would generally be properly provisioned, but resources for isolated garrisons in frontier areas were more limited.

Discerning patterns in disease incidence has direct utilitarian purpose for military forces, but in the nineteenth century officials were also looking to acquire as much information as they could about the vast expanse of continent across which the U.S. was expanding. In 1818 Louisiana was the only state that had been established west of the Mississippi River. In 1804 President Thomas Jefferson had dispatched Lewis and Clark to explore the Louisiana Purchase, with other expeditions exploring the west for decades. The medical reports compiled by the U.S. Army were part of this era of exploration. In addition to information on diagnoses, Surgeon General Lovell also required information on weather conditions from Army surgeons. “The influence of weather and climate upon diseases, especially epidemic, is perfectly well known,” declared Lovell. Collation of meteorological data could potentially validate the current miasmatic theory of disease, but such data also provided valuable information about the greater continent. Lovell was already publishing meteorological data in 1826, with more data published in 1840. ^2 , 3^

Monthly medical and meteorological reports continued to be required by the Army for decades in the nineteenth century. Compliance by surgeons seems high. Lovell's successor, Thomas Lawson, continued publishing health and meteorological data through 1860, ^4 , 5^ but routine reporting of weather data to Army headquarters was disrupted by the Civil War.

There was little need for redundant reports from units in the same place during the Civil War. Both health and weather condition reporting were often consolidated at a higher headquarters. A report, *Sickness and Mortality of the Army during the First Year of the War* , covering July 1861-June 1862, published by Surgeon General Joseph Barnes, mentions monthly reporting and strongly implies that medical officers were not being punctual nor accurate with their reports. ^6^ Recently volunteered doctors unfamiliar with U.S. Army reporting practices, lacking the discipline of regular officers to submit monthly reports, likely had lower compliance rates (George Wunderlich, email communication, Oct. 2024).

In the decades following the Civil War, Army reports on sickness could be both truthful and useless simultaneously. Before the acceptance of germ theory, few diseases could be differentiated. Regardless of diagnosis, there were few effective medicines, so an accurate or inaccurate diagnosis (in modern terms) made little impact on treatment or outcomes.

The Surgeon General's annual report to the Secretary of War, which was conveyed to Congress, would typically detail the number of admissions to hospital per thousand, as a broad indicator of force health. In 1884, diseases began to be grouped in the Surgeon General's report **(Figures 1a** **-** **1c)** . ^7^ During the ensuing decade, statistical comparisons became routine. In 1887 disease reports were further divided by geographic region. In 1888 diseases began being numbered—the predecessor of International Classification of Diseases codes—and by 1890 there was enough international agreement that the U.S. Army could compare its morbidity and mortality experience with foreign forces. By 1895, the Army was reporting its data based on the “diseases of the international nosological table.” ^8^

**FIGURE 1a.**
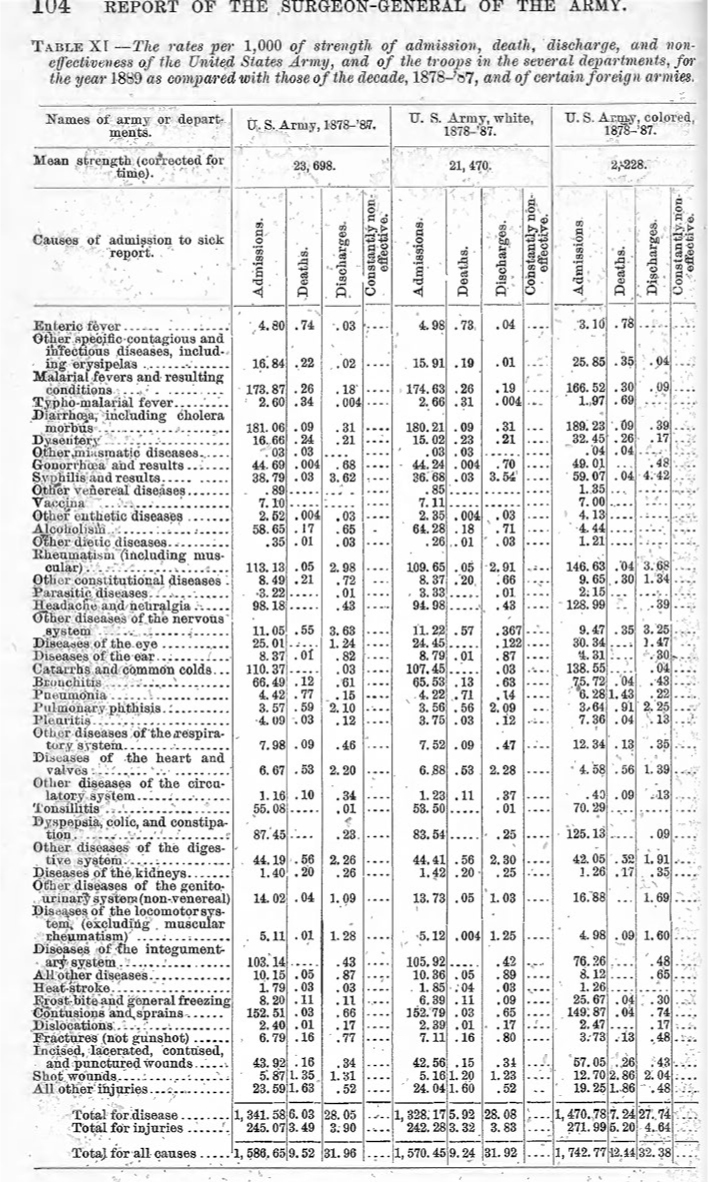
The Army interrogated its data to determine rates of incidence over time, regional rates, any racial differences, and establish international comparisons.

**FIGURE 1b.**
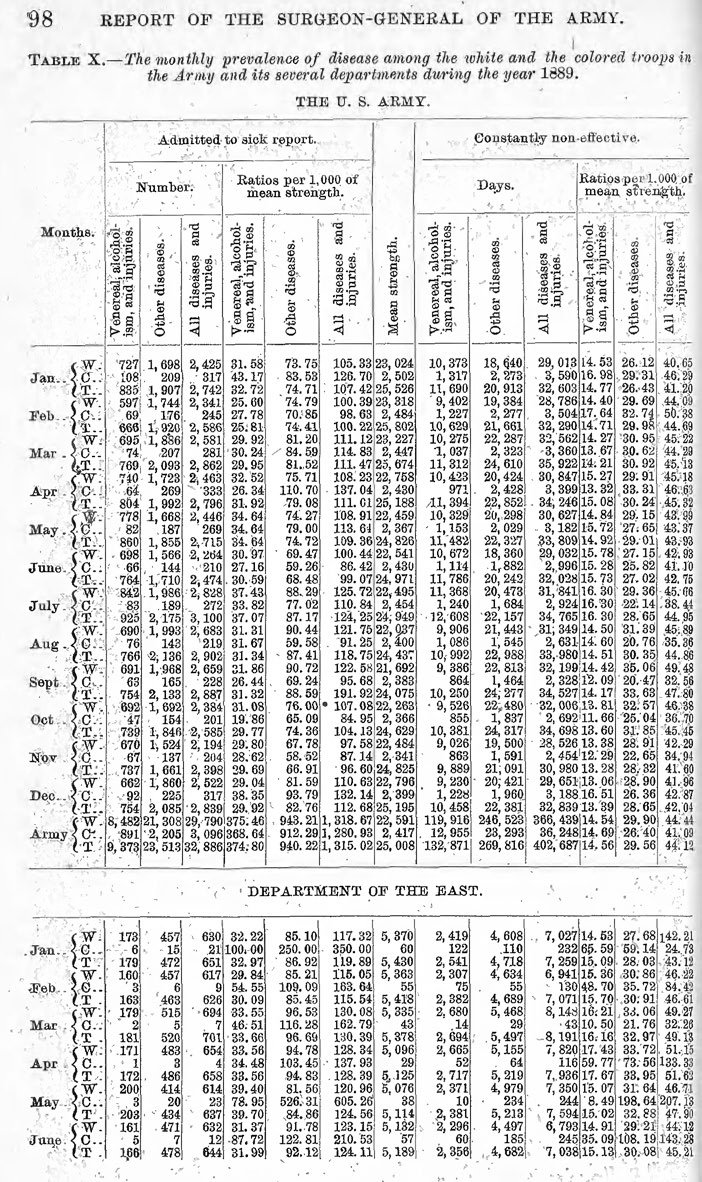
The Army further stratified its data to understand seasonal patterns in disease incidence and severity, for the force as a whole and regionally (not shown).

**FIGURE 1c.**
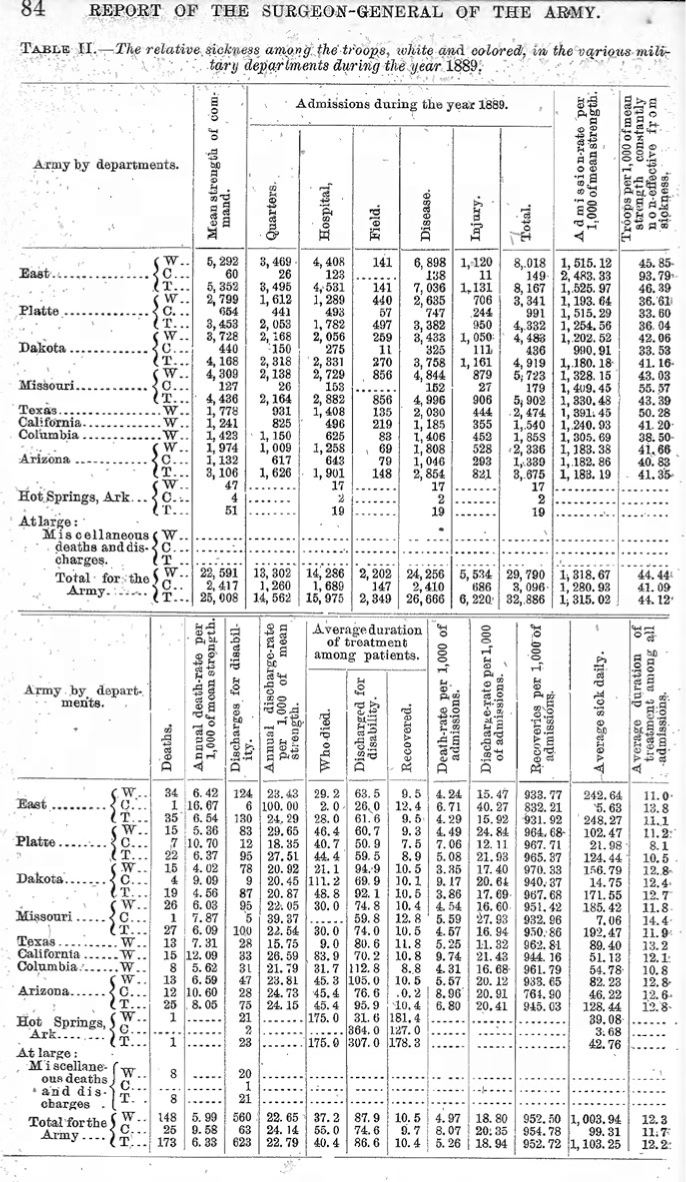
The Army also examined its data to evaluate disease burden quantity and severity by U.S. region.

By the close of the nineteenth century, the electric telegraph and railroads were widespread, allowing not only information but material to flow quickly. Patients as well as extra medical personnel could be moved, if necessary. A degree of local surveillance of conditions occurred, with alarming data rapidly reported to the Surgeon General's Office in Washington.

The Spanish-American War (April-December 1898) was the first major conflict fought by the U.S. in the era of germ theory. Disease was a significant problem during the war, with outbreaks of typhoid within the U.S. and malaria and yellow fever infecting troops in the Caribbean and South Pacific. By that time, the essentials of public health practice were being taught at the Army Medical School, now the Walter Reed Army Institute of Research, which was established in 1893.

Typhoid was endemic in the U.S. but became a scandal due to outbreaks at numerous Army encampments. A typhoid outbreak at Camp Thomas, outside Chattanooga, Tennessee, in 1898, was serious enough for Surgeon General George M. Sternberg to send a research team—of Walter Reed, Victor Vaughan, and Edward Shakespeare—to investigate it. Neither the groundbreaking scientific research by that trio, nor the statistics of the outbreak were published quickly: Statistics were still published within annual reporting, and the team's research was not published until 6 years later, in 1904, after Reed's and Shake-speare's deaths. ^9^

From 1913 until 1918, the Army Medical Department published a medical bulletin at uneven intervals, with 11 issues published in 6 years, apparently more a means of publishing research that was too long to be published in article form. By early 1918 the bulletin was focused solely on reconstruction, the term during that period for rehabilitation, anticipating wounded American soldiers' departure from the military hospital system and return to civilian life. ^10^ The 1918 iteration of the Army bulletin lasted only 4 issues, ending in late May, after which the Army apparently only published annual reports, either for internal or external audiences.

The following year, in December 1919 a twice-monthly newsletter, *Medico-Military Review* , began disseminating “information bearing upon the problems of disease control.” ^11^ Produced by the Division of Laboratories and Infectious Diseases, the *Review* was intended for internal audiences but was mailed to civilians who requested it. In the wake of the influenza pandemic, disease was a more salient topic for the Army, and the Military Intelligence Division of the General Staff was in close contact with the Surgeon General's Office about epidemics, and the Chief of Staff of the Army was briefed weekly on communicable diseases. ^12^

The *Medico-Military Review* published for over 2 years, until the advent of the *Army Medical Bulletin* in 1922. While there was mention of a “Medico-Military Review Section” of the *Bulletin* , there is no evidence such a section manifested. ^13^ For the next 2 decades, until World War II, surveillance data were available internally for outbreak responses, and annual data were published, but with no greater frequency.

After the first full year of combat in World War II, in 1943 the Army began publishing the *Monthly Progress Report* , which included a medical section. The medical section soon became lengthy enough that it was published separately, although still titled as part of the *Monthly Progress Report* . The medical section of the *Report* included surveillance information in addition to short articles on medical experiences in particular battles and campaigns, as well as particular diseases. In wartime this medical information had a security classification, albeit the second lowest.

After the war, the *Monthly Progress Report* transitioned to *Health of the Army* , and continued publishing monthly **(Figure 2a)** . It is unclear what element of the Surgeon General's Office produced this postwar reporting, which amalgamated data reported to, and analyzed by, several sections. In the early 1950s the report ceased publishing articles that provided medical analysis, other than occasional “diseases of special interest,” and *Health of the Army* published purely surveillance data, over-seen by the Patient Administration Systems and Biostatistics Activity. *Health of the Army* ceased publication at the end of 1988, but its last analytical article had appeared decades earlier, and it had evolved into a proto-dashboard of data—but published monthly, printed, and mailed.

**FIGURE 2a.**
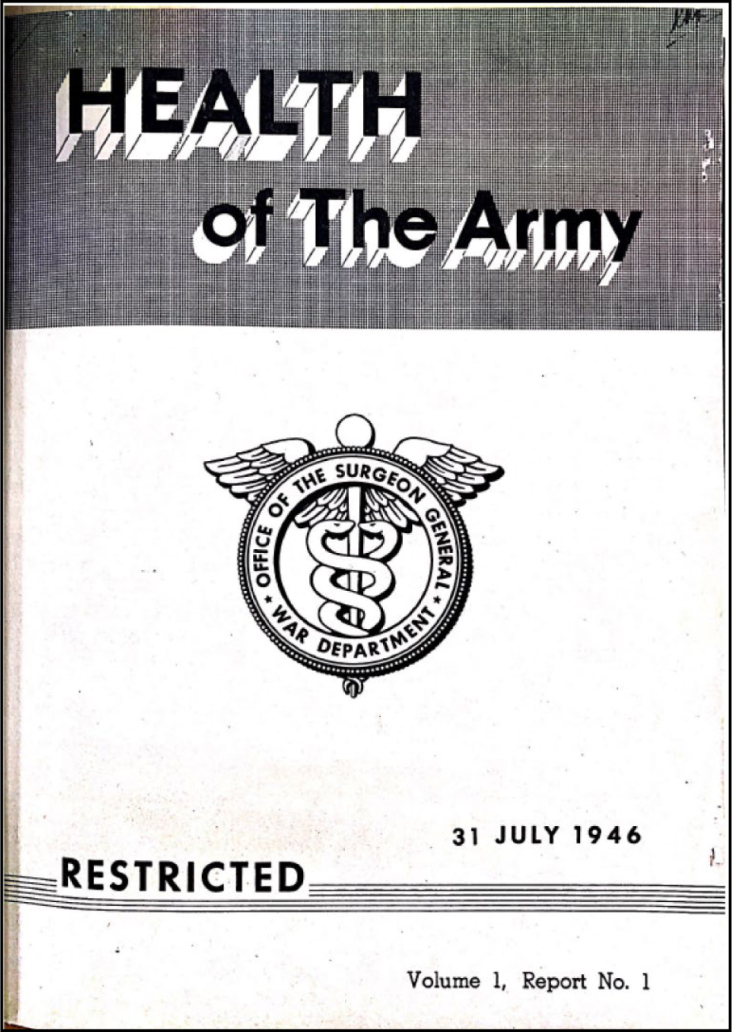
Inaugural Issues of and *Health of the Army* (1946) and *Statistics of Navy Medicine* (1945)

During the 20th century the U.S. Navy published some medical data in annual reports and published a monthly *Naval Medical Bulletin* , from 1907 to 1949, which was replaced by *Statistics of Navy Medicine* , from 1945 until 1989 **(Figure 2b)** (Andre Sobocinski, email communication, Oct. 2024). The U.S. Air Force published some medical data in annual reports, in addition to internal disease surveillance (Joseph Frechette, email communication, Oct. 2024).

**FIGURE 2b.**
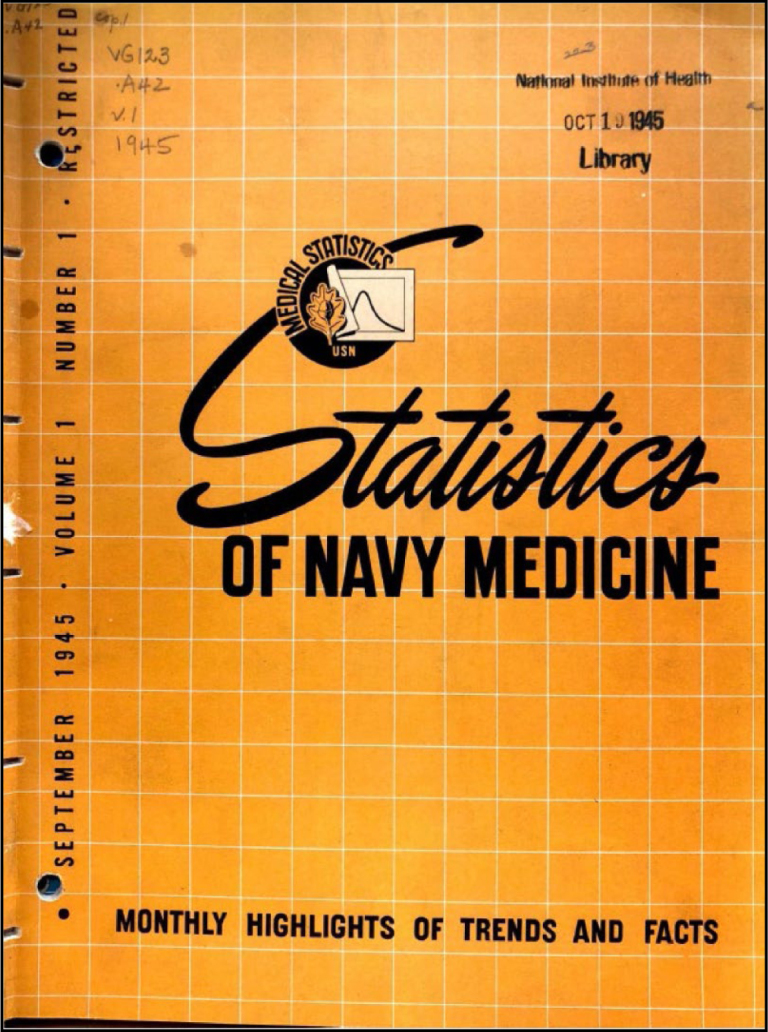
Inaugural Issues of and *Health of the Army* (1946) and *Statistics of Navy Medicine* (1945)

The nature of medical surveillance changes continuously, but the value in gathering, analyzing, and disseminating available data is constant. Different types and forms of data have been useful during different time cycles, whether for responding to a particular outbreak or investigating disease patterns over years. Throughout history, publishing and distribution patterns have been dictated by the relative rapidity of available data transmission. Whatever the limitations of current medical understanding, data collection and analysis, and available publishing and distribution, the U.S. military has consistently utilized the best data it could collect, analyze, and disseminate, to not only protect the health and lives of its personnel, but to improve current medical knowledge and practice, in addition to advancing scientific discovery.
